# Supplementary material for: Symmetry preference in shapes, faces, flowers and landscapes
Source: PeerJ. 2019 Jun 17;7:e7078. doi: 10.7717/peerj.7078 (PMC6585942; doi:10.7717/peerj.7078)
Supplement: Supplemental Information 1 [file peerj-07-7078-s001.docx]

# Symmetry preference in shapes, faces, flowers and landscapes

Supplementary Materials

# Marco Bertamini^1^, Giulia Rampone^2^, Alexis Makin^1^, Andrew Jessop^3^

# ^1^Department of Psychological Sciences, University of Liverpool, Liverpool, UK

# ^2^School of Psychology, University of Liverpool, Liverpool, UK

# ^3^Max Planck Institute for Psycholinguistics, Nijmegen, The Netherlands

In the main study we used images of faces (males and females), abstract shapes (smooth and angular), flowers, and landscapes. For each item we had two versions, one was called the original and the other was modified to have perfect bilateral symmetry. Each observer rated only one version of a given item. We analysed rating of beauty and ratings of salience (how clear and obvious was the subjective perception of symmetry).

Here in this supplementary study we report a replication with important changes to the stimuli and the procedure. The main differences between the main and the supplementary studies were: (a) In the new design we included the greyscale images as well as full colour images of the items. This allowed us to see whether the effects of shape (symmetry of the images) would remain the same when an additional factor is present that makes the images more variable and possibly more engaging. (b) As we only had greyscale images for the faces (from Rhodes et al. 1998) we did not include faces in this new study and we also excluded smooth abstract shapes. This was convenient to prevent the number of trials becoming too large given that including colour already doubled the number of stimuli. (c) In the original study we were very keen to ensure that observers would not see both versions of an item (original and perfect symmetry). In the supplementary study, instead, we included both versions so as to have the comparison within subjects.

## Methods

### Participants

Twenty-five individuals (5 male, 20 female) took part in the study and were recruited from the University of Liverpool student community. The age range was 18-21 and three were left handed.

### Stimuli

There were nine sets of ten pairs of images. For each set, ten images were symmetrical items (vertical bilateral reflection) and ten images were the original (non perfectly symmetrical items). There were five categories: angular shapes, smooth shapes, flowers, landscapes. All images were in greyscale. We used a black background and the images appeared within a square region of 500 pixels, except for the landscapes that had a height of 500 pixels and a width of 800 pixels.

Abstract shapes: There were two categories of abstract shapes, one was the original polygons and we refer to these stimuli as Angular (ten pairs in Greyscale and ten pairs in Colour). A second set was created by using a cubic spline that made the contour smooth. We refer to this set as Smooth (ten pairs in Greyscale and ten pairs in Colour).

Flowers: the flower stimuli were from taken from a study by Hůla and Flegr (2016). Starting from the original images we manipulated symmetry to obtain pairs of images (ten pairs in Greyscale and ten pairs in Colour). One of the pair had the original image of the flower and the other had perfect bilateral symmetry.

Landscapes: Images of outdoor scenes were downloaded from the internet. Starting from the original images we manipulated vertical symmetry to obtain pairs of images (ten pairs in Greyscale and ten pairs in Colour). As for the other categories, one of the pair had the original image of the landscape and the other had perfect bilateral symmetry.

### Procedure

The experiment had approval from The Health and Life Sciences Research Ethics Committee (Psychology, Health and Society) at the University of Liverpool (Ref: Bertamini: 0540). All participants were given information and signed a consent form before the start of the study.

Each participant was tested individually in a quiet room. They completed two phases always in the same order. First participants rated the beauty of each image using a mouse to control a rating scale (1 = not at all, 10 = very beautiful). Second, they used a similar rating scale to rate how “clear” and “salient” the symmetry was in the images (1 = not at all, 10 = very salient).

### Analysis

The analysis used generalised linear mixed-effects models implemented in the *lme4* package (Bates, Mächler, Bolker, & Walker, 2015) in *R* version 3.3.3 (R Core Team, 2018). The dependent measures of these analyses were subjective ratings of beauty or symmetry. A standardisation procedure was applied to these scores, which involved subtracting the mean from the individual ratings, and then dividing each score by the standard deviation. The mean and standard deviations used to standardise the scores were derived from each individual participant rather than the whole sample. All models included symmetry (symmetric/original) as an effect coded fixed factor. The model was simplified until convergence was reached where necessary. The marginal and conditional $R^{2}$ effect sizes are reported as measures of the variance explained by the model with the random effect structure included (conditional $R^{2}$) and excluded (marginal $R^{2}$) from the calculation.

## Results

### Preference (Beauty Ratings)

**

*Figure 1S.* Dumbbell plots to show the mean beauty ratings for each category in colour and in greyscale. Error bars represent the standard error adjusted for the model random effects structure.

Figure 1S shows the mean ratings for beauty for each of the three categories. This Figure can be compared with Figure 3 in the paper.

The model that converged for the abstract stimuli contained symmetry and colour as fully-crossed random slopes for subject. This model showed a significant main effect of colour ($\beta$ = -0.1002, *SE* = 0.048, $\chi^{2}$ = 4.13, *p* = .042) but not of symmetry ($\beta$ = -0.1002, *SE* = 0.048, $\chi^{2}$ = 4.13, *p* = .042) , and there was no interaction between colour and symmetry ($\beta$ = -0.0081, *SE* = 0.013, $\chi^{2}$ = 0.41, *p* = .523). The model accounted for 4.05% of the variance in the data without the random-effects, but 47.74% when they were included ($R_{m}^{2}$ = 0.0405; $R_{c}^{2}$ = 0.4774).

The model that converged for the flower stimuli contained colour as random slopes for subject. This model showed a significant main effect of colour ($\beta$ = -0.252, *SE* = 0.0267, $\chi^{2}$ = 39.31, *p* < .001) and of symmetry ($\beta$ = 0.0375, *SE* = 0.015, $\chi^{2}$ = 6.21, *p* = .013), and no interaction between colour and symmetry ($\beta$ = -0.0019, *SE* = 0.015, $\chi^{2}$ = 0.02, *p* = .901). This accounted for 13.44% of the variance in the data without the random-effects, but 37.12% when they were included ($R_{m}^{2}$ = 0.1344; $R_{c}^{2}$ = 0.3712).

The model that converged for the landscape stimuli contained symmetry and colour, but not their interaction, as random slopes for subject. The model showed a main effect of colour ($\beta$ = -0.3026, *SE* = 0.0356, $\chi^{2}$ = 35.01, *p* < .001) and of symmetry ($\beta$ = 0.1283, *SE* = 0.0383, $\chi^{2}$ = 15.54, *p* < .001), but no interaction between colour and symmetry ($\beta$ = -0.0028, *SE* = 0.016, $\chi^{2}$ = 0.03, *p* = .860). This accounted for 19.28% of the variance in the data without the random-effects, but 37.91% when they were included ($R_{m}^{2}$ = 0.1928; $R_{c}^{2}$ = 0.3791).

### Salience Ratings

**

*Figure 2S.* Dumbbell plots to show the mean salience ratings of each stimuli type in colour and greyscale. Error bars represent the standard error adjusted for the model random effects structure.

The model that converged for abstract stimuli contained symmetry as random slopes for subject. This model showed a significant main effect of symmetry ($\beta$ = -0.8647, *SE* = 0.0476, $\chi^{2}$ = 67.24, *p* < .001), but not of colour ($\beta$ = 0.008, *SE* = 0.0132, $\chi^{2}$ = 0.21, *p* = .643), and no interaction between colour and symmetry ($\beta$ = 0.0068, *SE* = 0.0132, $\chi^{2}$ = 0.26, *p* = .608). This model accounted for 66% of the variance in the data without the random-effects, but 76.8% when they were included ($R_{m}^{2}$ = 0.66; $R_{c}^{2}$ = 0.768).

The model that converged for flower stimuli contained colour as random slopes for subject. This model showed a significant main effect of symmetry ($\beta$ = -0.5977, *SE* = 0.015, $\chi^{2}$ = 1075.78, *p* < .001), but not of colour ($\beta$ = -0.0305, *SE* = 0.0163, $\chi^{2}$ = 2.38, *p* = .123), and no interaction of colour and symmetry ($\beta$ = -0.0197, *SE* = 0.015, $\chi^{2}$ = 1.72, *p* = .190). The model accounted for 49.92% of the variance in the data without the random-effects, but 52.99% when they were included ($R_{m}^{2}$ = 0.4992; $R_{c}^{2}$ = 0.5299).

The model that converged for landscape stimuli contained symmetry as random slopes for subject. This model showed significant main effects of colour ($\beta$ = -0.0219, *SE* = 0.0105, $\chi^{2}$ = 8.36, *p* = .004) and of symmetry ($\beta$ = -1.0542, *SE* = 0.0587, $\chi^{2}$ = 66.77, *p* < .001). There was a trend for the interaction between colour and symmetry ($\beta$ = 0.0204, *SE* = 0.0105, $\chi^{2}$ = 3.79, *p* = .052). The model accounted for 77.36% of the variance in the data without the random-effects, but 88.51% when they were included ($R_{m}^{2}$ = 0.7736; $R_{c}^{2}$ = 0.8851).

### Relationship between Beauty and Salience

**

*Figure 3S.* Scatterplots to show the relationship beauty and salience ratings split by whether the stimuli were in the original or symmmetrical version, and in colour or greyscale.

The maximal model that converged contained salience as random slopes for trial. All random effects for subject needed to be removed to achieve convergence. The model contained symmetry and salience as centered continuous predictors, and colour (colour/greyscale) as an effect coded fixed factor. Stimulus type was not included due to issues with overfitting the data.

The model observed significant main effects of salience ($\beta$ = 0.2887, *SE* = 0.0238, $\chi^{2}$ = 25.73, *p* < .001), symmetry ($\beta$ = 0.2272, *SE* = 0.0257, $\chi^{2}$ = 143.79, *p* < .001), and colour ($\beta$ = 0.2034, *SE* = 0.0257, $\chi^{2}$ = 217.79, *p* < .001). Critically, there was also a significant interaction between symmetry and salience ($\beta$ = -0.2179, *SE* = 0.0232, $\chi^{2}$ = 86.28, *p* < .001), which reflected that salience had a different impact on beauty rating for the symmetrical than the original stimuli. This interaction was not influenced by colour; that is, there was no three-way interaction between salience, symmetry and colour ($\beta$ = -0.0331, *SE* = 0.0233, $\chi^{2}$ = 2.03, *p* = .154). Overall, the model explained 10.26% of the variance in the data without the random-effects, but 12.03% when they were included ($R_{m}^{2}$ = 0.1026; $R_{c}^{2}$ = 0.1203).

## Discussion

Human preference for symmetry and regularity is a well-known phenomenon. In this study we used a simple rating task with images that were manipulated to introduce perfect bilateral symmetry. For each stimulus there was a pair of images, one was the original and the other the symmetrical manipulation (perfect bilateral symmetry). In this study, unlike Experiment 1 in the paper, each observer saw both item from a given pair. We refer to the two as symmetrical and original, however, original stimuli did possess some degree of symmetry. The results highlight that the beauty of landscapes is evaluated differently from the other type of stimuli. We argue that this is due to the fact that beauty of symmetry is always evaluated within an object. Symmetry of layout is fundamentally different and does not contribute to perceived beauty. Despite this, once the landscape becomes a new type of stimulus, unlike any real landscape, then and only then salience of symmetry is positively correlated with ratings of beauty.
